# Change in physical activity and the risk of depressive symptoms in mid‐older aged adults

**DOI:** 10.1111/ggi.70033

**Published:** 2025-03-24

**Authors:** Yu‐Tai Liu, Chien‐Yu Lin, Yung Liao, Ai Shibata, Kaori Ishii, Koichiro Oka

**Affiliations:** ^1^ Graduate School of Sport Sciences Waseda University Tokorozawa Japan; ^2^ Department of Public Health College of Medicine, National Cheng Kung University Tainan Taiwan; ^3^ Faculty of Sport Sciences Waseda University Tokorozawa Japan; ^4^ Centre for Urban Transitions Swinburne University of Technology Melbourne Australia; ^5^ Graduate Institute of Sport, Leisure and Hospitality Management, National Taiwan Normal University Taipei Taiwan; ^6^ Faculty of Health and Sport Sciences University of Tsukuba Tokyo Japan

**Keywords:** changes in physical activity, depression, initial activity level, maintaining active, middle‐aged and older adults

## Abstract

**Aim:**

Middle‐age and older adults' physical activity participation changes may influence their depressive conditions. This study explores the associations between changes in physical activity levels over time and the likelihood of depressive symptoms based on a population‐based survey.

**Methods:**

This study included adults aged ≥50 years from the Taiwan Longitudinal Study in Aging (TLSA) survey. Physical activity levels from the 2003 and 2007 surveys were used to identify patterns of change across time. Multiple logistic regression was performed to examine the associations between changes in physical activity and the risk of depressive symptoms. Additional analyses were conducted to assess if the associations of change patterns varied by individuals' baseline physical activity levels.

**Results:**

Of the 3439 participants, 12.9% newly developed a high risk of depression over 4 years. Compared with those with constant physical activity levels, adults with decreased physical activity levels showed a significantly higher risk of depressive symptoms at follow‐up (odds ratio = 1.59, 95% confidence interval: 1.23–2.06). The deteriorative association was most pronounced among individuals who had high physical activity levels at baseline but later reduced their activity (odds ratio = 2.01, 95% confidence interval: 1.48–2.72) compared with those who kept highly active. No significant association was observed among individuals who reported increased physical activity during the study period.

**Conclusion:**

These findings highlight the importance of middle‐aged and older adults staying physically active and avoiding reducing physical activity levels to prevent depression. **Geriatr Gerontol Int 2025; 25: 670–676**.

## Graphical abstract

- This study captured the aging population's integrated activity levels across time and investigated their association with depression risk.
- The findings indicate that decreasing physical activity over time is linked to increased depressive symptoms, suggesting that regular activity and staying active may help prevent depression in older adults.

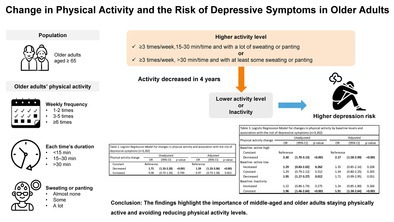

## Introduction

Mental disorders, including depressive disorders, anxiety disorders, and bipolar disorder, remained among the leading causes of disease burden between 1990 and 2019.^1^ It has been estimated that over 950 million people have mental disorders globally,^2^ with an escalating impact on healthcare and societal burdens. Between 1990 and 2019, the proportion of global disability‐adjusted life‐years due to mental disorders increased from 3.1% to 4.9%.^1^ Depressive disorders are among the most common mental disorders, and their presence will increase the risk of self‐harm and suicide,^3^ which in turn causes large medical expenditures and productivity losses.^4^ Given this disease burden, there is an imperative to better understand potential prevention strategies that reduce the risk of depression, which includes a focus on the modifiable behaviors involved in daily life.

Physical activity has various physiological effects, such as increasing endorphin and monoamine levels and reducing stress hormone cortisol. In addition, physical activity can divert an individual from negative thoughts by focusing on movements or social interactions.^5^, ^6^ Evidence has indicated that engagement in a certain amount of physical activity is linked to a reduced risk of depression in adult populations.^7^, ^8^ A recent meta‐analysis of cohort studies reported that people with higher or increased frequency and amount of physical activity were associated with a lower risk of depression compared with those at lower or decreased levels.^9^ However, participation in physical activity varies across different life stages. While physical activity levels generally decline with age, exercise, and leisure‐time physical activity may increase following retirement, highlighting the complexity of activity patterns across life stages.^10^ For the aging population, evidence has indicated that muscle mass begins to decline about 50 years of age, with the loss becoming more pronounced after 60 years of age.^11^ This physiological feature may impact their physical activity levels, making it essential to understand changes in their physical activity when exploring its relationship with depression. A variety of measurement types have been used to assess changes in physical activity when investigating their associations with depression risk. For instance, a study found a decrease in the weekly frequency (times per week) of vigorous‐intensity physical activity associated with a higher risk of depressive symptoms in women only.^12^ Other studies showed that those who remained sufficiently active (≥150 min/week) or became sufficiently active (e.g., increased activity level to meet physical activity recommendations) could lower the development of depression.^13^, ^14^ Most studies have investigated the association between variations (increased, maintained, and decreased) among older adults, typically defining individuals' physical activity levels based only on daily duration, single‐time intensity, or weekly frequency.^15^, ^16^, ^17^, ^18^ There is a scarcity of research that integrates multiple factors of weekly physical activity (such as weekly frequency, duration, or intensity each time) to examine changes in physical activity levels and the risk of depression in aging adults. Furthermore, it is unclear whether differences in baseline physical activity levels could account for variations in the association between physical activity change patterns and subsequent depression. Thus, this study investigates the associations between changes in physical activity level and depression risk among middle‐aged and older adults aged ≥50 years and examines whether the association differed by their baseline physical activity status.

## Methods

### Study population

Data were from the 2003 (baseline for this study) and 2007 (follow‐up for this study) Taiwan Longitudinal Study on Aging (TLSA), which are quadrennial national surveys. There have been nine waves of the TLSA to date since the first wave was implemented in 1989. Participants were recruited in 1989 using a three‐stage stratified cluster approach. Approximately one‐sixth (56 of 311) non‐Indigenous townships were randomly selected in the first stage, and lis (i.e., the smallest administrative unit in Taiwan) were sampled within the selected townships in the second stage. Residents aged ≥60 years from the selected lis were recruited. The sampling methods have been previously described in detail.^19^ To expand the participants to those during early‐stage aging (aged ≥50 years), additional cohorts of participants aged 50–66 were recruited in 1996, 2003, and 2015. The sample's age in the TLSA surveys ranged from ≥50 years (no upper age limit). Participants were interviewed in person by trained interviewers to collect their demographic characteristics and health‐related conditions. The response rate for each wave of the TLSA survey ranged from 70.7% to 92.1%.^20^ In the 2003 survey, 5369 participants provided the measures of physical activity. The analytic sample was 3439 participants, after excluding individuals lost to be contacted (due to death or other reasons; *n* = 1007), who did not report depression data in the baseline and follow‐up survey (*n* = 403), resided in long‐term care institutions (*n* = 14), provided missing data for covariates (*n* = 5) were disabled at baseline (indicated by the Activities of Daily Living Scale >0; *n* = 117), and individuals with higher depressive risk at the 2003 survey (*n* = 384). The study received ethical approval from the National Taiwan Normal University Ethics Committee (approval number 202103HM011).

### Depressive symptoms

Depressive symptoms were measured in 2003 and 2007 surveys using the 10‐item Center of Epidemiological Studies‐Depression (CES‐D‐10) scale.^21^ The 2007 records were used to define the outcome. The CES‐D‐10 is a modified version of the 20‐item CES‐D scale that has been shown to have adequate reliability and validity in Chinese populations,^22^ comprising two sets of symptoms: the presence of negative mood (e.g., “I felt that everything I did was an effort” and “I felt depressed”) and the positive mood (e.g., “I was happy” and “I enjoyed life”). Each item of the CES‐D‐10 was used to assess the frequency of experiencing each symptom in the past week on a four‐point scale (0 = never, 1 = rarely, 2 = sometimes, 3 = often or chronically). The scores for items assessing the presence of positive mood were reversely coded. The summed score of CES‐D‐10 ranged between 0 and 30. A higher score indicates severe depressive symptoms and a higher tendency to be depressed. A score of ≥10 was categorized as a higher risk of depressive symptoms.^23^

### Physical activity

Physical activity levels were extracted from the 2003 and 2007 TLSA questionnaires. Participants were asked to report the frequency, duration of each time, and intensity of their physical activity. Following the previous studies that have integrated these three components of physical activity,^24^, ^25^ each order was scored: (i) frequency, categorized as “inactive (score 0),” “1–2 times/week (1),” “3–5 times/week (2),” and “≥6 times/week (3)”; (ii) minutes spent each time, with options of “<15 min (score 1),” “15–30 min (2),” and “>30 min (3)”; and (iii) sweating or panting status, rated as “Almost none (score 1),” “some (2),” and “a lot (3).” Subsequently, the physical activity score was calculated by multiplying the scores from these three components with a range of 0–18. Participants' self‐reported physical activities for the 2003 and 2007 surveys were respectively categorized into “inactive” (physical activity score 0), “active‐low” (physical activity score 1–11), and “active‐high” (≥12) to represent the level difference of physical activity from diverse component combinations.

Changes in physical activity level between baseline (2003) and follow‐up surveys (2007) were categorized into three broad categories, consisting of nine subcategories, as follows:

1. To move to a category with a lower level (decreased): (i) from “active‐low” to “inactive”; (ii) from “active‐high” to “active‐low”; (iii) from “active‐high” to “inactive”;
2. To move to a category with the same level (constant): (iv) constant inactive; (v) constant “active‐low”; (vi) constant “active‐high”;
3. Moved to a category with a higher level (increased): (vii) from “inactive” to “active‐low”; (viii) from “inactive” to “active‐high”; and (ix) from “active‐low” to “active‐high.”

### Covariates

Baseline sociodemographic characteristics (i.e., sex, age, educational level, marital status, employment, and economic satisfaction), lifestyle factors (smoking, alcohol use, and body mass index [BMI]), chronic illness, social participation, and baseline depressive symptoms were included as covariates. BMI was calculated using self‐reported weight in kilograms divided by height in meters squared. We categorized BMI (kg/m^2^) into underweight (<18.5), normal (18.5–23.9), overweight (24–26.9), and obesity (≥27).^26^ Chronic illness was assessed by asking whether the participants had ever received physicians' diagnoses of any hypertension, diabetes, heart disease, or cancer (yes vs. no). Social participation was assessed by any involvement in groups such as volunteers, religious, political, or community (none vs. any).

### Analyses

The number and percentage of participants' characteristics were demonstrated. Chi‐squared tests were used to compare differences in these characteristics between participants' changes in physical activity levels. We examined the collinearity between the covariates. Multivariate logistic regression models were used to examine the associations of change in physical activity level (decreased, constant, and increased physical activity during the study period) with depression risk, estimating the odds ratios (ORs) and their 95% confidence intervals (CIs). Subsequent analyses of physical activity change patterns by baseline physical activity level were also performed (decreased physical activity from “active‐high” or “active‐low” level, constant inactive or “active‐low” or “active‐high,” and increased physical activity from “inactivity” or “active‐low”). The reference groups were assigned to those with constant physical activity levels and those with constant high activity levels over the study period. Unadjusted and fully adjusted models were both performed. A sensitivity analysis, including and adjusting for participants with high levels of depressive risk at baseline, was conducted to examine the robustness of the findings. In addition, an age‐stratified analysis (50–64 years vs. ≥65 years) was performed, as physical activity levels and depression risk vary with age.^27^ All analyses were performed using SAS version 9.4 (SAS Institute Inc., Cary, NC, USA). Statistical significance was set at *p* < 0.05.

## Results

Table 1 shows participants' characteristics. There were 52.6% men and 43.7% aged 50–59 years. The majority of the participants reported constant levels of physical activity over the study period, followed by one‐fourth of them reporting increased levels of physical activity. In total, 12.9% of participants developed a high risk of depression over the study period. Older adults who were relatively older (≥70 years), with lower education levels, without alcohol use, and with chronic illness were more likely to decrease physical activity levels over the study period. Detailed characteristics by subcategory of change in physical activity levels were shown in Table S1.

**Table 1:** Baseline characteristics of participants by changes in physical activity levels (*n* = 3439)

		Total	Decreased	Constant	Increased	*p*‐value
		*n* (%)	*n* (%)	*n* (%)	*n* (%)	
Overall		3439 (100)	654 (19.02)	2053 (59.69)	732 (21.29)	
Sex						0.055
Male		1808 (52.57)	321 (49.08)	1112 (54.16)	375 (51.23)	
Female		1631 (47.43)	333 (50.92)	941 (45.84)	357 (48.77)	
Age (years)						<0.001
50–59		1501 (43.65)	243 (37.16)	870 (42.38)	388 (53.01)	
60–69		900 (26.17)	170 (25.99)	552 (26.89)	178 (24.32)	
≥70		1038 (30.18)	241 (36.85)	631 (30.74)	166 (22.68)	
Education level						<0.001
No formal education		573 (16.66)	144 (22.02)	317 (15.44)	112 (15.3)	
Elementary school		1630 (47.4)	320 (48.93)	949 (46.23)	361 (49.32)	
High school		868 (25.24)	127 (19.42)	555 (27.03)	186 (25.41)	
College or above		368 (10.7)	63 (9.63)	232 (11.3)	73 (9.97)	
Marital status						0.199
Married		2632 (76.53)	483 (73.85)	1584 (77.16)	565 (77.19)	
Not married		807 (23.47)	171 (26.15)	469 (22.84)	167 (22.81)	
Occupational status						<0.001
Non‐employed		2163 (62.9)	471 (72.02)	1300 (63.32)	392 (53.55)	
Employed		1276 (37.1)	183 (27.98)	753 (36.68)	340 (46.45)	
Economic satisfaction						0.035
Satisfied		1449 (42.13)	276 (42.2)	887 (43.21)	286 (39.07)	
Fair		1204 (35.01)	233 (35.63)	724 (35.27)	247 (33.74)	
Dissatisfied		786 (22.86)	145 (22.17)	442 (21.53)	199 (27.19)	
Social participation						0.150
No		1864 (54.2)	362 (55.35)	1086 (52.9)	416 (56.83)	
Yes		1575 (45.8)	292 (44.65)	967 (47.1)	316 (43.17)	
Body mass index (kg/m^2^)						0.560
Underweight		166 (4.83)	31 (4.74)	94 (4.58)	41 (5.6)	
Normal		1619 (47.08)	318 (48.62)	971 (47.3)	330 (45.08)	
Overweight		1017 (29.57)	176 (26.91)	616 (30)	225 (30.74)	
Obesity		637 (18.52)	129 (19.72)	372 (18.12)	136 (18.58)	
Smoking						0.475
No		2669 (77.61)	509 (77.83)	1604 (78.13)	556 (75.96)	
Yes		770 (22.39)	145 (22.17)	449 (21.87)	176 (24.04)	
Alcohol use						0.040
No		2274 (66.12)	460 (70.34)	1340 (65.27)	474 (64.75)	
Yes		1165 (33.88)	194 (29.66)	713 (34.73)	258 (35.25)	
Hypertension						0.010
No		2439 (70.92)	442 (67.58)	1449 (70.58)	548 (74.86)	
Yes		1000 (29.08)	212 (32.42)	604 (29.42)	184 (25.14)	
Diabetes						0.014
No		3069 (89.24)	563 (86.09)	1845 (89.87)	661 (90.3)	
Yes		370 (10.76)	91 (13.91)	208 (10.13)	71 (9.7)	
Heart diseases						0.001
No		2992 (87)	543 (83.03)	1790 (87.19)	659 (90.03)	
Yes		447 (13)	111 (16.97)	263 (12.81)	73 (9.97)	
Cancer						0.167
No		3364 (97.82)	635 (97.09)	2016 (98.2)	713 (97.4)	
Yes		75 (2.18)	19 (2.91)	37 (1.8)	19 (2.6)	
2007 depression risk						<0.001
Low		2996 (87.12)	535 (81.8)	1811 (88.21)	650 (88.8)	
High		443 (12.88)	119 (18.2)	242 (11.79)	82 (11.2)	

In the unadjusted models, those who reduced their levels of physical activity over the study period were associated with higher odds of depression risk at follow‐up (OR = 1.67; 95% CI: 1.31–2.11), compared with those reporting constant levels of physical activity (Table 2). The association was slightly attenuated after adjusting for all covariates (OR = 1.59; 95% CI: 1.23–2.06). No statistical evidence was found for those who increased physical activity levels over the study period.

**Table 2:** Logistic regression model for changes in physical activity and association with the risk of depressive symptoms (*n* = 3439)

Physical activity change	Unadjusted			Adjusted		
	OR	(95% CI)	*P*‐value	OR	(95% CI)	*P*‐value
Constant	Reference			Reference		
Decreased	**1.67**	**(1.31–2.11)**	**<0.001**	**1.59**	**(1.23–2.06)**	**<0.001**
Increased	0.94	(0.73–1.23)	0.672	0.97	(0.73–1.28)	0.813

*Note*: Adjusted models included sex, age, education level, marital status, occupational status, economic satisfaction, social participation, smoking, alcohol use, body mass index, chronic illness, hypertension, diabetes, heart disease, and cancer. Bold values refer to significant differences (*p* < 0.05).

Abbreviations: 95% CI, 95% confidence interval; OR, odds ratio.

Analyses of activity changes by baseline levels showed similar associations between changes in physical activity levels and depression risk (Table 3). Compared with those who maintained “active‐high” levels of physical activity, those who decreased their physical activity from “active‐high” levels (OR = 2.01, 95% CI: 1.48–2.72), “active‐low” levels (OR = 1.68, 95% CI: 1.02–2.79), and constantly inactive (OR = 1.93, 95% CI: 1.42–2.63) were associated with higher odds of depression risk after the adjustment for all covariates. No statistical evidence for associations was found between changes in physical activity levels and depression risk in other subcategories. Similar results of decreasing levels of physical activity associated with a higher risk of depression were found in sensitivity analyses (Tables S2 and S3). The age‐stratified analysis (Tables S4 and S5) indicated that among adults aged ≥65 years (*n* = 1439), decreasing physical activity over time was significantly associated with higher depression risk, regardless of baseline activity levels. For those aged 50–64 (*n* = 2000), the estimates were similar but not statistically significant, except for the deteriorative effect of reduced activity from the “active‐high” level and remaining inactive.

**Table 3:** Logistic Regression Model for changes in physical activity by baseline levels and association with the risk of depressive symptoms (*n* = 3439)

Physical activity change	Unadjusted			Adjusted		
	OR	(95% CI)	p‐value	OR	(95% CI)	p‐value
Baseline: active‐high						
Constant	Reference			Reference		
Decreased	**2.20**	**(1.64–2.94)**	**<0.001**	**2.01**	**(1.48–2.72)**	**<0.001**
Baseline: active‐low						
Increased	1.27	(0.83–1.96)	0.272	1.35	(0.87–2.11)	0.189
Constant	1.32	(0.83–2.11)	0.239	1.32	(0.81–2.16)	0.264
Decreased	**2.03**	**(1.26–3.27)**	**0.004**	**1.68**	**(1.02–2.79)**	**0.044**
Baseline: Inactivity						
Increased	1.20	(0.86–1.69)	0.299	1.24	(0.87–1.78)	0.235
Constant	**1.95**	**(1.47–2.58)**	**<0.001**	**1.93**	**(1.42–2.63)**	**<0.001**

*Note*: Adjusted models included sex, age, education level, marital status, occupational status, economic satisfaction, social participation, smoking, alcohol use, body mass index, hypertension, diabetes, heart disease, and cancer. Bold values refer to significant differences (*p* < 0.05).

Abbreviations: 95% CI, 95% confidence interval; OR, odds ratio.

## Discussion

This study investigated the associations of changes in levels of physical activity between 2003 and 2007 with depression risk in middle‐aged and older adults aged ≥50 years. Our results indicated that people who reduced their physical activity levels were associated with an increased risk of depressive symptoms compared with those who remained at constant levels, mainly in line with previous findings.^12^, ^15^, ^28^ Decreasing physical activity over time could lead to fewer opportunities to experience self‐esteem and social interaction through physical activity.^29^ Furthermore, the lack of participation in activity often contributes to diminished energy and motivation among aging adults, potentially exacerbating the depression risk associated with the decline in physical activity.^28^, ^30^, ^31^ The present study adds new insights by revealing age differences in the association between changes in physical activity and depression risk. Older adults (≥65 years) who decreased their activity from any levels of activity initially experienced more significant detrimental effects, while middle‐aged and early older adults (50–64 years) had weaker associations, suggesting the associations of reduced activity on increased depression risk may intensify with the aging process.

No associations were found between increased levels of physical activity and the risk of depression, which is inconsistent with previous findings that indicated increased physical activity was associated with a reduced risk of depression among middle‐aged and older adults.^7^, ^8^ The absence of a protective effect of physical activity on mental health may be attributed to aging‐related changes in functional capacity, which have been demonstrated to harm individuals' well‐being.^28^ Furthermore, it is important to note that this study lacked consideration of sedentary behaviors. Past studies indicated that people engaged in longer sedentary time were associated with a higher risk of depression.^32^, ^33^ It is plausible that the aging process and sedentary time, independently of physical activity time, may be associated with a higher risk of depression and offset potential positive effects on mental well‐being in later life. Future research is needed to take into account the 24‐h movement framework to examine the associations between behaviors and mental health.

Analyses of change patterns by baseline activity level revealed that the negative associations of change in physical activity levels with depression risk were particularly for those who dropped an “active‐high” level and reduced physical activity levels from “active‐low” to inactive for older adults age ≥65 years. These findings align with previous evidence of older adults indicating that decreasing physical activity can strengthen the association of higher depressive symptoms at follow‐up, whether the participant stayed in strenuous activity or had a lower activity level at baseline.^15^, ^28^ Furthermore, people with inactive levels demonstrate an elevation in incident depression compared with those participating in lower or even minimal physical activity.^9^, ^34^ The negative impact of reducing physical activity on depression risk may escalate when individuals become inactive during the follow‐up period.^35^ No significant association was observed in individuals aged 50–64 years for decreasing activity from “active‐low” levels, which could be explained by previous evidence reporting age‐related differences in physical activity and depression before and after 65 years.^27^ Nevertheless, the results still support the negative association of decreasing activity from a higher level and staying inactive with depression in aging adults. Overall, our findings underscore that keeping regular physical activity (exceeding 30 min, at least 3–5 times/week with sweating or panting) is an applicable practice not to develop depressive symptoms subsequently. In addition, it is crucial to avoid inactivity, particularly for people who have lowered their activity.

The strength of this study was the longitudinal design for data collection on physical activity and depressive symptoms. This study identified the physical activity levels based on clear dimensions of physical activity (weekly frequency, minutes each time, and experience of sweating or panting) and thus provided intuitive references. Several limitations of this study need to be acknowledged. The self‐reports of physical activity and depressive symptoms may raise concerns of recall bias and/or be affected by social desirability.^36^, ^37^ In addition, despite this study applying longitudinal data, the ascertain of physical activity levels change patterns and depression symptoms defined in the 2007 survey cannot rule out the possibility of reverse causality. Furthermore, there may be some residual confounding factors, such as sedentary behaviors and medication usage, that were not assessed in the TLSA survey.^38^ Future research is encouraged to include objective measures for physically active and sedentary behaviors (e.g., accelerometer) and depressive symptoms (e.g., physician's diagnosis). The TLSA surveys were conducted every 4 years, and thus we were unable to distinguish the changes in physical activity levels as well as the mental state during the study period.

In conclusion, the study findings emphasize that the aging population with reduced physical activity levels was more likely to develop depression, regardless of any level of regular physical activity initially. Our findings suggest it is important to develop sustainable strategies for promoting or sustaining physical activity levels with regular reminders such as messages and aftercare phone calls that prevent middle‐aged and older adults from decreasing their physical activity levels in their aging years.

## Disclosure statement

The authors declare no conflict of interest.

## Author contribution

Y.T.L. initiated the research idea and conducted the analysis. C.Y.L. and A.S. supervised the design and writing. All authors discussed the findings and contributed to the final manuscript.

## Funding

This study was not supported by any funding source.

## Supporting information


**TABLE S1.** Baseline characteristics of participants with physical activity change group by baseline levels (*n* = 3439).
**Table S2.** Logistic Regression Model for changes in physical activity and association with the risk of depressive symptoms with adjustment for baseline depression (*n* = 3823).
**Table S3.** Logistic Regression Model for changes in physical activity by baseline levels and association with the risk of depressive symptoms with adjustment for baseline depression (*n* = 3823).
**Table S4.** Age‐stratified Logistic Regression Model for changes in physical activity and association with the risk of depressive symptoms.
**Table S5.** Age‐stratified Logistic Regression Model for changes in physical activity by baseline levels and association with the risk of depressive symptoms.

## Data Availability

This study did not have a preregistration. The Taiwan Longitudinal Study in Aging survey data are not publicly available. Researchers can obtain the availability for study purposes by applying with the Health and Welfare Data Science Center, Ministry of Health and Welfare, Taiwan (https://dep.mohw.gov.tw/DOS/cp-5119-59201-113.html), or requesting from the corresponding author with the permission of the Ministry of Health and Welfare, Taiwan.
