# Supplementary material for: Change in physical activity and the risk of depressive symptoms in mid‐older aged adults
Source: Geriatr Gerontol Int. 2025 Mar 24;25(5):670–6. doi: 10.1111/ggi.70033 (PMC12055516; doi:10.1111/ggi.70033)
Supplement: Supplementary file 1 — TABLE S1. Baseline characteristics of participants with physical activity change group by baseline levels (n = 3439). Table S2. Logistic Regression Model for changes in physical activity and association with the risk of depressive symptoms with adjustment for baseline depression (n = 3823). Table S3. Logistic Regression Model for changes in physical activity by baseline levels and association with the risk of depressive symptoms with adjustment for baseline depression (n = 3823). Table S4. Age‐stratified Logistic Regression Model for changes in physical activity and association with the risk of depressive symptoms. Table S5. Age‐stratified Logistic Regression Model for changes in physical activity by baseline levels and association with the risk of depressive symptoms. [file GGI-25-670-s001.docx]

| Supplemental Table 1. Baseline characteristics of participants with physical activity change group by baseline levels (n=3,439) | | | | | | | | | | | |
| --- | --- | --- | --- | --- | --- | --- | --- | --- | --- | --- | --- |
| Physical activity changes | | Baseline: Inactivity | |  | Baseline: Active-low | | |  | Baseline: Active-high | | p-value |
|  |  | Constant | Increased |  | Decreased | Constant | Increased |  | Decreased | Constant |  |
| Overall | | 611 (17.77) | 482 (14.01) |  | 139 (4.04) | 200 (5.82) | 250 (7.27) |  | 515 (14.98) | 1242 (36.11) |  |
| Sex | |  |  |  |  |  |  |  |  |  | 0.010 |
|  | Male | 328 (53.68) | 251 (52.07) |  | 56 (40.29) | 94 (47) | 124 (49.6) |  | 265 (51.46) | 690 (55.56) |  |
|  | Female | 283 (46.32) | 231 (47.93) |  | 83 (59.71) | 106 (53) | 126 (50.4) |  | 250 (48.54) | 552 (44.44) |  |
| Age |  |  |  |  |  |  |  |  |  |  | <0.001 |
|  | 50-59 | 311 (50.9) | 250 (51.87) |  | 64 (46.04) | 111 (55.5) | 138 (55.2) |  | 179 (34.76) | 448 (36.07) |  |
|  | 60-69 | 162 (26.51) | 124 (25.73) |  | 36 (25.9) | 42 (21) | 54 (21.6) |  | 134 (26.02) | 348 (28.02) |  |
|  | ≥ 70 | 138 (22.59) | 108 (22.41) |  | 39 (28.06) | 47 (23.5) | 58 (23.2) |  | 202 (39.22) | 446 (35.91) |  |
| Education level | |  |  |  |  |  |  |  |  |  | <0.001 |
|  | No formal education | 121 (19.8) | 83 (17.22) |  | 36 (25.9) | 37 (18.5) | 29 (11.6) |  | 108 (20.97) | 159 (12.8) |  |
|  | Elementary school | 342 (55.97) | 248 (51.45) |  | 74 (53.24) | 73 (36.5) | 113 (45.2) |  | 246 (47.77) | 534 (43) |  |
|  | High school | 124 (20.29) | 112 (23.24) |  | 24 (17.27) | 66 (33) | 74 (29.6) |  | 103 (20) | 365 (29.39) |  |
|  | College or above | 24 (3.93) | 39 (8.09) |  | 5 (3.6) | 24 (12) | 34 (13.6) |  | 58 (11.26) | 184 (14.81) |  |
| Marital status | |  |  |  |  |  |  |  |  |  | 0.322 |
|  | Married | 486 (79.54) | 369 (76.56) |  | 99 (71.22) | 152 (76) | 196 (78.4) |  | 384 (74.56) | 946 (76.17) |  |
|  | Not married | 125 (20.46) | 113 (23.44) |  | 40 (28.78) | 48 (24) | 54 (21.6) |  | 131 (25.44) | 296 (23.83) |  |
| Occupational status | |  |  |  |  |  |  |  |  |  | <0.001 |
|  | Non-employed | 286 (46.81) | 257 (53.32) |  | 93 (66.91) | 113 (56.5) | 135 (54) |  | 378 (73.4) | 901 (72.54) |  |
|  | Employed | 325 (53.19) | 225 (46.68) |  | 46 (33.09) | 87 (43.5) | 115 (46) |  | 137 (26.6) | 341 (27.46) |  |
| Economic satisfaction | |  |  |  |  |  |  |  |  |  | <0.001 |
|  | Satisfied | 188 (30.77) | 180 (37.34) |  | 48 (34.53) | 71 (35.5) | 106 (42.4) |  | 228 (44.27) | 628 (50.56) |  |
|  | Fair | 225 (36.82) | 174 (36.1) |  | 56 (40.29) | 81 (40.5) | 73 (29.2) |  | 177 (34.37) | 418 (33.66) |  |
|  | Dissatisfied | 198 (32.41) | 128 (26.56) |  | 35 (25.18) | 48 (24) | 71 (28.4) |  | 110 (21.36) | 196 (15.78) |  |
| Social participation | |  |  |  |  |  |  |  |  |  | <0.001 |
|  | No | 373 (61.05) | 276 (57.26) |  | 73 (52.52) | 121 (60.5) | 140 (56) |  | 289 (56.12) | 592 (47.67) |  |
|  | Yes | 238 (38.95) | 206 (42.74) |  | 66 (47.48) | 79 (39.5) | 110 (44) |  | 226 (43.88) | 650 (52.33) |  |
| Body Mass Index, BMI | |  |  |  |  |  |  |  |  |  | 0.011 |
|  | Underweight | 38 (6.22) | 30 (6.22) |  | 6 (4.32) | 7 (3.5) | 11 (4.4) |  | 25 (4.85) | 49 (3.95) |  |
|  | Normal | 259 (42.39) | 206 (42.74) |  | 63 (45.32) | 87 (43.5) | 124 (49.6) |  | 255 (49.51) | 625 (50.32) |  |
|  | Overweight | 176 (28.81) | 149 (30.91) |  | 40 (28.78) | 64 (32) | 76 (30.4) |  | 136 (26.41) | 376 (30.27) |  |
|  | Obesity | 138 (22.59) | 97 (20.12) |  | 30 (21.58) | 42 (21) | 39 (15.6) |  | 99 (19.22) | 192 (15.46) |  |
| Smoking | |  |  |  |  |  |  |  |  |  | <0.001 |
|  | No | 419 (68.58) | 353 (73.24) |  | 106 (76.26) | 162 (81) | 203 (81.2) |  | 403 (78.25) | 1023 (82.37) |  |
|  | Yes | 192 (31.42) | 129 (26.76) |  | 33 (23.74) | 38 (19) | 47 (18.8) |  | 112 (21.75) | 219 (17.63) |  |
| Alcohol use | |  |  |  |  |  |  |  |  |  | 0.051 |
|  | No | 382 (62.52) | 304 (63.07) |  | 102 (73.38) | 128 (64) | 170 (68) |  | 358 (69.51) | 830 (66.83) |  |
|  | Yes | 229 (37.48) | 178 (36.93) |  | 37 (26.62) | 72 (36) | 80 (32) |  | 157 (30.49) | 412 (33.17) |  |
| Hypertension | |  |  |  |  |  |  |  |  |  | <0.001 |
|  | No | 468 (76.6) | 360 (74.69) |  | 95 (68.35) | 148 (74) | 188 (75.2) |  | 347 (67.38) | 833 (67.07) |  |
|  | Yes | 143 (23.4) | 122 (25.31) |  | 44 (31.65) | 52 (26) | 62 (24.8) |  | 168 (32.62) | 409 (32.93) |  |
| Diabetes | |  |  |  |  |  |  |  |  |  | 0.011 |
|  | No | 558 (91.33) | 442 (91.7) |  | 117 (84.17) | 185 (92.5) | 219 (87.6) |  | 446 (86.6) | 1102 (88.73) |  |
|  | Yes | 53 (8.67) | 40 (8.3) |  | 22 (15.83) | 15 (7.5) | 31 (12.4) |  | 69 (13.4) | 140 (11.27) |  |
| Heart diseases | |  |  |  |  |  |  |  |  |  | 0.004 |
|  | No | 546 (89.36) | 437 (90.66) |  | 115 (82.73) | 172 (86) | 222 (88.8) |  | 428 (83.11) | 1072 (86.31) |  |
|  | Yes | 65 (10.64) | 45 (9.34) |  | 24 (17.27) | 28 (14) | 28 (11.2) |  | 87 (16.89) | 170 (13.69) |  |
| Cancer | |  |  |  |  |  |  |  |  |  | 0.204 |
|  | No | 602 (98.53) | 473 (98.13) |  | 135 (97.12) | 198 (99) | 240 (96) |  | 500 (97.09) | 1216 (97.91) |  |
|  | Yes | 9 (1.47) | 9 (1.87) |  | 4 (2.88) | 2 (1) | 10 (4) |  | 15 (2.91) | 26 (2.09) |  |
| 2007 depression risk | |  |  |  |  |  |  |  |  |  | 0.011 |
|  | Low | 509 (83.31) | 429 (89) |  | 115 (82.73) | 176 (88) | 221 (88.4) |  | 420 (81.55) | 1126 (90.66) |  |
|  | High | 102 (16.69) | 53 (11) |  | 24 (17.27) | 24 (12) | 29 (11.6) |  | 95 (18.45) | 116 (9.34) |  |

| Supplemental Table 2. Logistic Regression Model for changes in physical activity and association with the risk of depressive symptoms with adjustment for baseline depression (n=3,823) | | | | | | | | | |  |
| --- | --- | --- | --- | --- | --- | --- | --- | --- | --- | --- |
| Changes in physical activity levels | |  | Unadjusted | | |  | Adjusted | | |  |
|  |  |  | OR | (95% CI) | p-value |  | OR | (95% CI) | p-value |  |
|  | Constant |  | Reference |  |  |  | Reference |  |  |  |
|  | Decreased |  | **1.62** | **(1.32-2.00)** | **<0.001** |  | **1.60** | **(1.28-2.00)** | **<0.001** |  |
|  | Increased |  | 1.04 | (0.84-1.29) | 0.725 |  | 0.98 | (0.77-1.24) | 0.857 |  |
| Notes: OR = odds ratio; 95% CI = 95% confidence interval. Adjusted models included sex, age, education level, marital status, occupational status, economic satisfaction, social participation, smoking, alcohol use, Body Mass Index, chronic illness, hypertension, diabetes, heart diseases, cancer, and baseline depressive risk. Bold values refer to significant differences (p-value < 0.05) | | | | | | | | | |  |
|  |  |  |  |  |  |  |  |  |  |  |

| Supplemental Table 3. Logistic Regression Model for changes in physical activity by baseline levels and association with the risk of depressive symptoms with adjustment for baseline depression (n=3,823) | | | | | | | | | |
| --- | --- | --- | --- | --- | --- | --- | --- | --- | --- |
| Changes in physical activity levels | |  | Unadjusted | | |  | Adjusted | | |
|  |  |  | OR | (95% CI) | p-value |  | OR | (95% CI) | p-value |
| Baseline: active-high | |  |  |  |  |  |  |  |  |
|  | Constant |  | Reference |  |  |  | Reference |  |  |
|  | Decreased |  | **2.17** | **(1.68-2.81)** | **<0.001** |  | **2.10** | **(1.60-2.76)** | **<0.001** |
| Baseline: active-low | |  |  |  |  |  |  |  |  |
|  | Increased |  | **1.57** | **(1.12-2.22)** | **0.011** |  | 1.43 | (0.98-2.09) | 0.067 |
|  | Constant |  | 1.18 | (0.78-1.79) | 0.442 |  | 1.16 | (0.74-1.82) | 0.525 |
|  | Decreased |  | **1.95** | **(1.28-2.97)** | **0.002** |  | **1.73** | **(1.10-2.72)** | **0.019** |
| Baseline: Inactivity | |  |  |  |  |  |  |  |  |
|  | Increased |  | 1.26 | (0.95-1.67) | 0.122 |  | 1.17 | (0.86-1.60) | 0.326 |
|  | Constant |  | **2.02** | **(1.60-2.57)** | **<0.001** |  | **1.84** | **(1.40-2.41)** | **<0.001** |
| Notes: OR = odds ratio; 95% CI = 95% confidence interval. Adjusted models included sex, age, education level, marital status, occupational status, economic satisfaction, social participation, smoking, alcohol use, Body Mass Index, hypertension, diabetes, heart diseases, cancer, and baseline depressive risk. Bold values refer to significant differences (p-value < 0.05) | | | | | | | | | |
|  |  |  |  |  |  |  |  |  |  |

| Supplemental Table 4. Age-stratified Logistic Regression Model for changes in physical activity and association with the risk of depressive symptoms | | | | | | | | | |  |
| --- | --- | --- | --- | --- | --- | --- | --- | --- | --- | --- |
| Changes in physical activity levels | |  | 50-64 years old (n=2,000) | | |  | ≥65 years old (n=1,439) | | |  |
|  |  |  | OR | (95% CI) | p-value |  | OR | (95% CI) | p-value |  |
|  | Constant |  | Reference |  |  |  | Reference |  |  |  |
|  | Decreased |  | 1.22 | (0.83-1.81) | 0.318 |  | **1.77** | **(1.27-2.46)** | **<0.001** |  |
|  | Increased |  | 0.90 | (0.62-1.30) | 0.565 |  | 1.05 | (0.69-1.58) | 0.832 |  |
| Notes: OR = odds ratio; 95% CI = 95% confidence interval. Adjusted for sex, age, education level, marital status, occupational status, economic satisfaction, social participation, smoking, alcohol use, Body Mass Index, chronic illness, hypertension, diabetes, heart disease, and cancer. Bold values refer to significant differences (p-value < 0.05) | | | | | | | | | |  |
|  |  |  |  |  |  |  |  |  |  |  |

| Supplemental Table 5. Age-stratified Logistic Regression Model for changes in physical activity by baseline levels and association with the risk of depressive symptoms | | | | | | | | | |
| --- | --- | --- | --- | --- | --- | --- | --- | --- | --- |
| Changes in physical activity levels | |  | 50-64 years old (n=2,000) | | |  | ≥65 years old (n=1,439) | | |
|  |  |  | OR | (95% CI) | p-value |  | OR | (95% CI) | p-value |
| Baseline: active-high | |  |  |  |  |  |  |  |  |
|  | Constant |  | Reference |  |  |  | Reference |  |  |
|  | Decreased |  | **1.89** | **(1.15-3.11)** | **0.013** |  | **2.17** | **(1.47-3.21)** | **<0.001** |
| Baseline: active-low | |  |  |  |  |  |  |  |  |
|  | Increased |  | 1.33 | (0.71-2.49) | 0.373 |  | 1.41 | (0.73-2.72) | 0.309 |
|  | Constant |  | 1.27 | (0.63-2.60) | 0.508 |  | 1.45 | (0.74-2.86) | 0.289 |
|  | Decreased |  | 1.29 | (0.59-2.85) | 0.527 |  | **2.19** | **(1.11-4.35)** | **0.025** |
| Baseline: Inactivity | |  |  |  |  |  |  |  |  |
|  | Increased |  | 1.25 | (0.76-2.07) | 0.380 |  | 1.24 | (0.73-2.11) | 0.432 |
|  | Constant |  | **2.10** | **(1.34-3.28)** | **0.001** |  | **1.79** | **(1.16-2.79)** | **0.010** |
| Notes: OR = odds ratio; 95% CI = 95% confidence interval. Adjusted models included sex, age, education level, marital status, occupational status, economic satisfaction, social participation, smoking, alcohol use, Body Mass Index, hypertension, diabetes, heart diseases, and cancer. Bold values refer to significant differences (p-value < 0.05) | | | | | | | | | |
|  |  |  |  |  |  |  |  |  |  |
